# EFFECTS OF FASTING STATUS ON BLOOD-BASED NEURODEGENERATIVE BIOMARKERS IN OLDER ADULTS

**DOI:** 10.1093/geroni/igae098.2621

**Published:** 2024-12-31

**Authors:** Christopher Engeland, Robert Tennyson, Molly Wright, Erik Knight, Orfeu Buxton, Jennifer Graham-Engeland

**Affiliations:** The Pennsylvania State University, University Park, Pennsylvania, United States; The Pennsylvania State University, University Park, Pennsylvania, United States; The Pennsylvania State University, University Park, Pennsylvania, United States; University of Colorado Boulder, Boulder, Colorado, United States; Pennsylvania State University, University Park, Pennsylvania, United States; Pennsylvania State University, University Park, Pennsylvania, United States

## Abstract

Blood-based neurodegenerative biomarkers hold vast potential to serve as early detectors of neuropathology, including Alzheimer’s disease. However, accurate biomarker assessment is critical and extraneous factors that may affect circulating concentrations need greater understanding. To determine the effects of fasting status on blood-based levels of neurodegenerative biomarkers, 30 older adults (Mage = 67.52; 70% women) fasted >8h prior to two laboratory visits. Each visit included 4 blood draws, spaced 1 hour apart. The visits (counterbalanced) were differentiated by a meal being provided after either the second or fourth blood draw. This design enabled examination of fasting versus post-prandial effects, accounting for circadian variation of biomarkers. Plasma levels of neurodegenerative biomarkers (amyloid β(Aβ) 42/40 ratio, neurofilament light chain (NfL), glial fibrillary acidic protein (GFAP), phosphorylated tau (pTau)181, pTau217) were quantified at each timepoint. Bayesian multilevel models (minimally informative priors, Stan MCMC sampler) compared all pre-prandial (6 timepoints) vs. post-prandial (2 timepoints) concentrations (covariates: time-of-blood-draw, visit order, age, gender, BMI). Results provided strong and very strong evidence for no changes in Aβ42/40 ratio and pTau181, respectively. However, there was very strong evidence that eating decreased GFAP levels, substantial evidence that eating decreased pTau217 levels, and weak evidence that eating decreased NfL levels. These findings indicate that plasma levels of some, but not all, neurodegenerative biomarkers are susceptible to changes due to eating. One implication is that eating should be controlled (e.g., via fasting) in studies incorporating GFAP and pTau217. Further research is needed for NfL, which exhibited less certain (weak) evidence of change.

